# Pilot study with pegylated liposomal doxorubicin for advanced or unresectable hepatocellular carcinoma

**DOI:** 10.1054/bjoc.2001.2149

**Published:** 2001-12

**Authors:** M Schmidinger, C Wenzel, G J Locker, F Muehlbacher, R Steininger, M Gnant, R Crevenna, A C Budinsky

**Affiliations:** Department of Internal Medicine I, Division of Clinical Oncology, Department of Surgery, Department of Physical Medicine and Rehabilitation, University of Vienna, Waehringer Guertel 18-20, Vienna, A-1090, Austria

**Keywords:** hepatocellular carcinoma, liposomal doxorubicin, chemotherapy, toxicity, survival

## Abstract

We performed a pilot-study on pegylated liposomal doxorubicin (PLD) for advanced hepatocellular carcinoma. Seventeen patients received 40 mg/m^2^ PLD intravenously every 4 weeks. A clinical benefit response was achieved in 50% (complete remission 7%, minor remission 7%, stable disease 36%). Toxicities were moderate. In view of these encouraging findings, further studies appear warranted. © 2001 Cancer Research Campaign http://www.bjcancer.com


*British Journal of Cancer* (2001) **85**(12), 1850–1852
© 2001 Cancer Research Campaign
doi: 10.1054/ bjoc.2001.2149, available online at http://www.idealibrary.com on IDEAL

http://www.bjcancer.com

### Pilot study with pegylated liposomal doxorubicin for advanced or unresectable hepatocellular carcinoma

**M Schmidinger[1], C Wenzel[1], GJ Locker[1], F Muehlbacher[2], R Steininger[2], M Gnant[2], R Crevenna[3], AC Budinsky[1] and GG Steger[1]**

[1]Department of Internal Medicine I, Division of Clinical Oncology; [2]Department of Surgery; and [3]Department of Physical Medicine and Rehabilitation, University of Vienna, Waehringer Guertel 18-20 A-1090 Vienna, Austria

**Summary** We performed a pilot-study on pegylated liposomal doxorubicin (PLD) for advanced hepatocellular carcinoma. Seventeen patients received 40 mg/m$^2$ PLD intravenously every 4 weeks. A clinical benefit response was achieved in 50% (complete remission 7%, minor remission 7%, stable disease 36%). Toxicities were moderate. In view of these encouraging findings, further studies appear warranted. © 2001 Cancer Research Campaign http://www.bjcancer.com

**Keywords**: hepatocellular carcinoma; liposomal doxorubicin; chemotherapy; toxicity; survival

Surgical resection is the only treatment with the potential for cure in hepatocellular carcinoma (HCC). In the case of unresectable tumour, regional intra-arterial treatment has become the treatment of choice, leading to response rates of 55 to 90% (Onohara et al, 1988; Sasaki et al, 1997; Fujimoto et al, 1985; Konno et al, 1983; Beppu et al, 1991). However, for patients with extrahepatic disease, systemic treatment might be the only therapeutical option.

Among systemic treatment modalities for advanced HCC, doxorubicin has been one of the most widely used and most active agents (Falkson et al, 1978, 1984; Lai et al, 1988). However, considering both, the modest response rates of less than 20% and the pronounced treatment related toxicities, doxorubicin is not considered a major breakthrough in the treatment of HCC (Mathurin et al, 1998).

In contrast to free doxorubicin, pegylated liposomal doxorubicin (PLD) – a stealth liposome formula of doxorubicin – ensures longer circulation time and preferential accumulation in tumour tissue, thus resulting in both, higher therapeutic efficacy and reduced toxicity (Gabizon et al, 1986; Gabizon and Martin, 1997). Whereas PLD appears to be superior to doxorubicin in the murine model (Zou et al, 1993), little is known on the therapeutic efficacy and safety of PLD in the treatment of HCC, particularly when administered intravenously (i.v.).

The aim of this pilot study was to test the efficacy and toxicity of i.v. PLD in patients with advanced HCC. Since with the standard FDA-approved protocol of 50 mg/m$^2$ (Muggia et al, 1997; Ranson et al, 1997) significant skin toxicities and mucositis have been reported, our protocol was to administer 40 mg/m$^2$ only.

*Received 24 April 2001*
*Revised 9 August 2001*
*Accepted 18 September 2001*

*Correspondence to:* M Schmidinger

### PATIENTS AND METHODS

### Patients

Seventeen patients (10 male, 7 female) with metastatic or unresectable HCC entered the study. The patients characteristics are given in Table 1. No patient had previously received adjuvant or palliative chemotherapy. Eligibility criteria consisted of an expected survival of at least 3 months, age between 19 and 80 years, a Karnofsky performance status of at least 80%, adequate bone marrow function with white blood cell count above 3000/µl, platelet count above 75 000/µl, haemoglobin above

**Table 1** Patient characteristics

| Characteristic | *n* = Number of patients |
|---|---|
| Entered | 17 |
| Evaluable toxicity | 17 |
| Evaluable response | 14 |
| Age, years | |
| Median | 63 |
| Range | 29–76 |
| Sex | |
| Male | 10 |
| Female | 7 |
| Liver cirrhosis | 13 |
| Child A/B | 10/3 |
| Chronic hepatitis B/C | 3/5 |
| Treatment prior to disease progression | |
| Liver transplantation | 3 |
| Resection | 3 |
| Cryotherapy | 3 |
| Interferon-alpha | 5 |
| Tumour Stage (UICC 1997) | |
| III B | 3 |
| IV A | 7 |
| IV B | 6 |
| Extrahepatic Disease | 6 |
| Visceral | 1 |
| Non-visceral | 5 |

10 mg% and organ functions within normal ranges. All patients had to give informed consent according to institutionals regulations.

Three patients became ineligible for response analysis. One patient died before the first tumour-reassessment from a therapy-unrelated endocarditis. Two patients were withdrawn from the study due to the misinterpretation of an incompatibility reaction after an undue preparation of the infusion. Finally, there were 14 patients eligible for the analysis of efficacy whereas all patients were eligible for final toxicity evaluation.

### Treatment plan and patient evaluation

PLD (Caelyx, licensed by Aesca Ges.m.b.H. Badener Straße 23, A-2514 Traiskirchen, Austria) was diluted in 500 ml glucose 5% and administered intravenously every 28 days at a dose of 40 mg/m$^2$. The prophylactic anti-emetic treatment consisted of 8 mg ondansetron and 4 mg dexamethasone given intravenously. A minimum of three courses of treatment had to be given before the first tumour reassessment. In case of remission or stabilization of disease, additional treatment courses were administered until disease progression.

In case of WHO grade III toxicity, the dose of PLD was reduced by 25%. Any WHO grade 4 toxicity led to discontinuation of therapy and withdrawal of the patient from treatment.

### Statistical analysis

Survival curves were calculated and plotted according to the method of Kaplan and Meier. Statistical comparisons of survival were done using Wilcoxon's test. *P* values below 0.05 were considered significant.

### RESULTS

### Toxicity

A total of 81 courses (median 3 per patient, range 1–24) were given to the patients. WHO grade III myelotoxicities included leucopenia, anaemia and thrombopenia in 12%, 6% and 6% of the patients, respectively. No cardiotoxicity was observed as assessed by physical symptoms and electrocardiography. No patient suffered from nausea or vomiting. Two patients experienced bronchospasm immediately after initiation of PLD-infusion. These events were first misinterpreted as a hypersensitivity reaction. In retrospect, this reaction was felt to be an incompatibility reaction to an undue preparation of the infusion. The line was not as required by the manufacture filled with glucose 5% but filled with normal saline. As a result of this misinterpretation these two patients were withdrawn from the drug regimen.

### Response

Response was assessed by computed tomography or magnetic resonance imaging. Objective tumour remissions were observed in 2 out of 14 patients (14%) (CR $n$ = 1 (7%), MR $n$ = 1 (7%) with a response duration of 24+ months both. The CR was verified by liver biopsy. Five patients (36%) experienced stable disease (SD) with a median duration of 6 months (range 3–11 months). Seven (50%) patients progressed under this treatment.

Alpha-fetoprotein (AFP) serum levels in the patient achieving CR decreased during the treatment from 76572 kU/L to a normal range (i.e. below 7 kU/L). In the patients with SD, AFP decreased from 38585 kU/l to 3027 kU/L ($n$ = 1), remained within the normal range ($n$ = 1), or increased ($n$ = 3) from a median of 332 (range; 14–384) up to 1101 (range; 42–2160) kU/L, respectively.

### Survival

At the time of analysis, the median time of follow-up is 27 months (range 10–44 months). The overall survival of all 17 patients was median 12 months (range 1–27 + months). The survival of patients with clinical benefit response (CR, MR or SD) and PD was 13 and 6 months, respectively. The two patients achieving objective remissions (CR and MR) are alive both 27 months after diagnosis of advanced disease without any signs of disease progression.

### DISCUSSION

The major finding of this study is that i.v. PLD is effective in terms of long-lasting objective remissions (OR) (14%) and long-lasting disease stabilizations (36%) in some patients with advanced HCC. At the time of analysis, the two patients achieving OR were alive 27 months since diagnosis of unresectable HCC. For the patient with CR, a liver transplantation is scheduled now.

To the best of our knowledge, only two authors have reported on their experience with i.v. PLD for HCC, with one case report showing encouraging results (Hong et al, 2000) and one phase II study showing neither OR nor SD (Halm et al, 2000).

Since our patient population appears comparable to those of the Halm-study in terms of age, WHO performance status and tumour stage, the different outcome (clinical benefit response 50% in our study versus neither OR nor SD in the Halm trial) might be explained by the dose-regimen. In our study all patients received 40 mg/m$^2$ PLD, whereas the dose in the Halm-trial was in the majority (75%) of the cycles 30 mg/m$^2$ only.

In our study, patients with SD showed only a trend toward a prolonged survival when compared to patients with PD. However, these data should be interpreted cautiously. First, the size of our study is too small to detect any statistical significant survival difference between these sub-groups. Second, as recently shown, the median survival of patients with HCC varies from 2.6 to 15.2 months dependent upon several prognostic features, such as portal vein thrombosis, tumour-mass, AFP-value and so on (Schoniger-Hekele et al, 2001). Therefore, one could argue that our patients might belong to a prognostic favourable sub-group. Third, from our small trial it remains unclear as to whether the survival length of our patients achieving SD is due to the specific antitumour treatment or might have been achieved with best supportive care alone. Whereas the superiority of a specific antitumour treatment over best supportive care has been demonstrated for other highly aggressive tumours (Ahlgren, 1996; Scheithauer et al, 1999; Hoffman and Glimelius, 1998; Burris et al, 1997), meta-analyses from randomized trials using doxorubicin for HCC did not reveal any survival benefit (Mathurin et al, 1998; Simonetti et al, 1997). However, these data were generated from the treatment with the free drug doxorubicin. The liposomal form, which was used in the present trial, might have higher therapeutic efficacy even in a highly chemoresistant tumour such as HCC.

A further observation of this pilot-study was the favourable toxicity-profile of PLD. Myelotoxicity was generally moderate.

© 2001 Cancer Research Campaign 

Similar favourable results were reported from studies using 45–50 mg/m$^2$ PLD in patients with advanced sarcoma (Judson et al, 2001), malignant mesothelioma (Baas et al, 2000), ovarian cancer (Gordon et al, 2000) and breast cancer. Higher myelotoxicities occurred only at dose levels beyond 60 mg/m$^2$ (Ranson et al, 1997).

In contrast to the moderate myelotoxicity, palmar-plantar erythrodysesthesia (PPE) was reported to be a major adverse event in these studies (Judson et al, 2001; Baas et al, 2000; Muggia et al, 1997). In accordance with the findings of Ranson et al, who found PPE to be greatly reduced at a dose of 45 mg/m$^2$ PLD, the lack of PPE in our trial might be explained by the dose of 40 mg/m$^2$.

As no patient suffered from nausea and vomiting in our own study and those from other investigators, PLD seems not to be particularly emetogenic. This is even true for PLD-trials performed without prophylactic anti-emetics (Halm et al, 2000; Toma et al, 2000). The reason for including a prophylactic anti-emetic treatment in our protocol was the lack of personal experience and of sufficient clinical data on PLD at the time when our trial was initiated. In view of our own results and the findings of other investigators, we now strongly support that PLD, when given as monotherapy, does not require the routine co-administration of anti-emetics.

Finally, since none – and this is true even for the patient receiving 24 courses of PLD – did experience any cardiotoxicity, PLD can be considered safe and feasible in an outpatient-setting.

We conclude that i.v. PLD can result in objective tumour remissions and long-lasting disease stabilization in patients with metastatic or unresectable HCC. Due to the lack of appropriate sized studies, the role of PLD in advanced HCC is not definitely defined yet.

### REFERENCES

Ahlgren JD (1996) Chemotherapy for pancreatic cancer. *Cancer* **78**(**Suppl** 3): 654–663

Baas P, van Meerbeeck J, Groen H, Schouwink H, Burgers S, Daamen S and Giaccone G (2000) Caelyx in malignant mesothelioma: a phase II EORTC study. *Ann Oncol* **11**: 697–700

Beppu T, Ohara C, Yamaguchi Y, Ichihara T, Yamanaka S, Katafuchi S, Ikei S, Mori K, Fukushima S and Nakano M (1991) A new approach to chemoembolization for unresectable hepatocellular carcinoma using aclarubicin microspheres in combination with cisplatin suspended in iodized oil. *Cancer* **68**: 2555–2560

Burris HA, Moore MJ, Andersen J, Green MR, Rothenberg ML, Modiano MR, Cripps MC, Portenoy RK, Storniolo AM, Tarassoff P, Nelson R, Dorr FA, Stephens CD and Von Hoff DD (1997) Improvements in survival and clinical benefit with gemcitabine as first-line therapy for patients with advanced pancreas cancer: a randomized trial. *J Clin Oncol* **15**: 2403–2413

Falkson G, Moertel CG, Lavin P, Pretorius FJ and Carbone PP (1978) Chemotherapy studies in primary liver cancer. A prospective randomized clinical trial. *Cancer* **42**: 2149–2156

Falkson G, MacIntyre J, Shutt AJ, Coetzer B, Johnson LA, Simson IW and Douglass HO Jr (1984) Neocarzinostatin versus mAMSA or doxorubicin in hepatocellular carcinoma. *J Clin Oncol* **2**: 581–584

Fujimoto S, Miyazaki M, Endoh F, Takahashi O, Okui K and Morimoto Y (1985) Biodegradable Mitomycin C microspheres given intra-arterially for inoperable hepatic cancer. *Cancer* **56**: 2404–2410

Gabizon A, Meshorer A and Barenholz Y (1986) Comparative long term study of the toxicities of free and liposome-associated doxorubicin in mice after intravenous administration. *J Natl Cancer Inst* **77**: 459–466

Gabizon A and Martin F (1997) Polyethylene glycol-coated (pegylated) liposomal doxorubicin. Rationale for use in solid tumors. *Drugs* **54**(**Suppl** 4): 15–21

Gordon AN, Granai CO, Rose PG, Hainsworth J, Lopez A, Weissman C, Rosales R and Sharpington T (2000) Phase II study of liposomal doxorubicin in platinum- and paclitaxel-refractory epithelial ovarian cancer. *J Clin Oncol* **18**: 3093–3100

Halm U, Etzrodt G, Schiefke I, Schmidt F, Witzigmann H, Mossner J and Berr F (2000) A phase II study of pegylated liposomal doxorubicin for the treatment of advanced hepatocellular carcinoma. *Ann Oncol* **11**: 113–114

Hoffman K and Glimelius B (1998) Evaluation of benefit of chemotherapy in patients with upper gastrointestinal cancer. *Acta Oncol* **37**: 651–659

Hong RL, Tseng YJ and Chang FH (2000) Pegylated liposomal doxorubicin in treating a case of advanced hepatocellular carcinoma with severe hepatic dysfunction and pharmacokinetic study. *Ann Oncol* **11**: 349–353

Judson I, Radford JA, Harris M, Blay JY, van Hoesel Q, le Cesne A, van Oosterom AT, Clemons MJ, Kamby C, Hermans C, Whittaker J, Donato di Paola E, Verweij J and Nielsen S (2001) Randomised phase II trial of pegylated liposomal doxorubicin in the treatment of advanced or metastatic soft tissue sarcoma: a study by the EORTC Soft Tissue and Bone Sarcoma group. *Eur J Cancer* **37**: 870–877

Konno T, Maeda H, Iwai K, Tashiro S, Maki K, Morinaga T, Mochinaga M, Hiraoka T and Yokoyama I (1983) Effect of arterial administration of high-molecular-anticancer agents SMANC with lipid lymphographic agent on hepatoma: a preliminary report. *Eur J Cancer Clin Oncol* **19**: 1053–1065

Lai C-L, Wu P-C, Chan GC-B, Lok AS and Lin HJ (1988) Doxorubicin versus no antitumor therapy in inoperable hepatocellular carcinoma. A prospective randomized trial. *Cancer* **62**: 479–483

Mathurin P, Rixe O, Carbonell N, Bernard B, Cluzel P, Bellin MF, Khayat D, Opolon P and Poynard T (1998) Review article: Overview of medical treatments in unresectable hepatocellular carcinoma-an impossible meta-analysis? *Aliment Pharmacol Ther* **12**

Muggia FM, Hainsworth JD and Jeffers S (1997) Phase II study of liposomal doxorubicin in refractory ovarian cancer: antitumor activity and toxicity modification by liposomal encapsulation. *J Clin Oncol* **15**: 987–993

Onohara S, Kobayashi H, Itoh Y and Shinohara S (1988) Intra-arterial cis-platinum infusion with sodium thiosulfate protection and angiotensin-II induced hypertension for treatment of hepatocellular carcinoma. *Acta Radiologica* **29**: 197–202

Ranson MR, Carmichael J, O'Byrne K, Stewart S, Smith D and Howell A (1997) Treatment of advanced breast cancer with sterically stabilized liposomal doxorubicin: results of a multicenter phase II trial. *J Clin Oncol* **15**: 3185–3191

Sasaki Y, Imacka S and Kasugai H (1997) A new approach to chemoembolisation therapy for hepatoma using ethiodized oil, cisplatin, gelatin sponge. *Cancer* **60**: 1194–1203

Scheithauer W, Kornek GV, Raderer M, Hejna M, Valencak J, Miholic J, Kovats E, Lang F, Funovics J, Bareck E and Depisch D (1999) Phase II trial of gemcitabine, epirubicin and granulocyte colony-stimulating factor in patients with advanced pancreatic adenocarcinoma. *Br J Cancer* **80**: 1797–1802

Schoniger-Hekele M, Muller C, Kutilek M, Oesterreicher C, Ferenci P and Gangl A (2001) Hepatocellular carcinoma in Central Europe: prognostic features and survival. *Gut* **48**: 103–109

Simonetti RG, Liberati A, Angiolini C and Pagliaro L (1997) Treatment of hepatocellular carcinoma: A systematic review of randomized controlled trials. *Ann Oncol* **8**: 117–136

Toma S, Tucci A, Villani G, Carteni G, Spadini N and Palumbo R (2000) Liposomal doxorubicin (Caelyx) in advanced pretreated soft tissue sarcomas: a phase II study of the Italian Sarcoma Group (ISG). *Anticancer Res* **20**: 485–491

Zou Y, Horikoshi Y, Kasagi T, Gu X and Perez-Soler R (1993) Organ distribution and antitumor activity of free and liposomal doxorubicin injected into the hepatic artery. *Cancer Chemother Pharmacol* **31**: 313–318

 *© 2001 Cancer Research Campaign*
